# Supplementary material for: Resolving the full spectrum of human genome variation using Linked-Reads
Source: Genome Res. 2019 Apr;29(4):635–45. doi: 10.1101/gr.234443.118 (PMC6442396; doi:10.1101/gr.234443.118)
Supplement: Supplemental Material [file supp_gr.234443.118_Supplemental_longranger-2.2.zip › longranger-2.2.tar.bz2!/lr2.2-public/lib/stan/cmdstan/stan/doc/api/html/stack__alloc_8hpp_source.html]

Stan: src/stan/memory/stack\_alloc.hpp Source File


|  |  |
| --- | --- |
|  | Stan  2.5.0  probability, sampling & optimization |


- Main Page
- Related Pages
- Namespaces
- Classes
- Files

- File List
- File Members

All Classes Namespaces Files Functions Variables Typedefs Enumerations Enumerator Friends Macros Pages

- src
- stan
- memory

stack\_alloc.hpp

Go to the documentation of this file.

1 #ifndef STAN\_\_MEMORY\_\_STACK\_ALLOC\_HPP

2 #define STAN\_\_MEMORY\_\_STACK\_ALLOC\_HPP

3

4 #include <cstdlib>

5 #include <cstddef>

6 #include <sstream>

7 #include <stdexcept>

8 #if defined(\_MSC\_VER)

9  #include <msinttypes.h> // Microsoft Visual Studio lacks compliant stdint.h

10 #else

11  #include <stdint.h> // FIXME: replace with cstddef?

12 #endif

13 #include <vector>

14 #include <stan/meta/likely.hpp>

15

16 namespace stan {

17

18  namespace memory {

19

31  template <typename T>

32  bool is\_aligned(T\* ptr, unsigned int bytes\_aligned) {

33  return (reinterpret\_cast<uintptr\_t>(ptr) % bytes\_aligned) == 0U;

34  }

35

36

37  namespace {

38  const size\_t DEFAULT\_INITIAL\_NBYTES = 1 << 16; // 64KB

39

40

41  // FIXME: enforce alignment

42  // big fun to inline, but only called twice

43  inline char\* eight\_byte\_aligned\_malloc(size\_t size) {

44  char\* ptr = static\_cast<char\*>(malloc(size));

45  if (!ptr) return ptr; // malloc failed to alloc

46  if (!is\_aligned(ptr,8U)) {

47  std::stringstream s;

48  s << "invalid alignment to 8 bytes, ptr="

49  << reinterpret\_cast<uintptr\_t>(ptr)

50  << std::endl;

51  throw std::runtime\_error(s.str());

52  }

53  return ptr;

54  }

55  }

56

76  class stack\_alloc {

77  private:

78  std::vector<char\*> blocks\_; // storage for blocks, may be bigger than cur\_block\_

79  std::vector<size\_t> sizes\_; // could store initial & shift for others

80  size\_t cur\_block\_; // index into blocks\_ for next alloc

81  char\* cur\_block\_end\_; // ptr to cur\_block\_ptr\_ + sizes\_[cur\_block\_]

82  char\* next\_loc\_; // ptr to next available spot in cur

83  // block

84  // next three for keeping track of nested allocations on top of stack:

85  std::vector<size\_t> nested\_cur\_blocks\_;

86  std::vector<char\*> nested\_next\_locs\_;

87  std::vector<char\*> nested\_cur\_block\_ends\_;

88

89

98  char\* move\_to\_next\_block(size\_t len) {

99  char\* result;

100  ++cur\_block\_;

101  // Find the next block (if any) containing at least len bytes.

102  while ((cur\_block\_ < blocks\_.size()) && (sizes\_[cur\_block\_] < len))

103  ++cur\_block\_;

104  // Allocate a new block if necessary.

105  if (unlikely(cur\_block\_ >= blocks\_.size())) {

106  // New block should be max(2\*size of last block, len) bytes.

107  size\_t newsize = sizes\_.back() \* 2;

108  if (newsize < len)

109  newsize = len;

110  blocks\_.push\_back(eight\_byte\_aligned\_malloc(newsize));

111  if (!blocks\_.back())

112  throw std::bad\_alloc();

113  sizes\_.push\_back(newsize);

114  }

115  result = blocks\_[cur\_block\_];

116  // Get the object's state back in order.

117  next\_loc\_ = result + len;

118  cur\_block\_end\_ = result + sizes\_[cur\_block\_];

119  return result;

120  }

121

122  public:

123

133  stack\_alloc(size\_t initial\_nbytes = DEFAULT\_INITIAL\_NBYTES) :

134  blocks\_(1, eight\_byte\_aligned\_malloc(initial\_nbytes)),

135  sizes\_(1,initial\_nbytes),

136  cur\_block\_(0),

137  cur\_block\_end\_(blocks\_[0] + initial\_nbytes),

138  next\_loc\_(blocks\_[0]) {

139  if (!blocks\_[0])

140  throw std::bad\_alloc(); // no msg allowed in bad\_alloc ctor

141  }

142

149  ~stack\_alloc() {

150  // free ALL blocks

151  for (size\_t i = 0; i < blocks\_.size(); ++i)

152  if (blocks\_[i])

153  free(blocks\_[i]);

154  }

155

168  inline void\* alloc(size\_t len) {

169  // Typically, just return and increment the next location.

170  char\* result = next\_loc\_;

171  next\_loc\_ += len;

172  // Occasionally, we have to switch blocks.

173  if (unlikely(next\_loc\_ >= cur\_block\_end\_))

174  result = move\_to\_next\_block(len);

175  return (void\*)result;

176  }

177

184  inline void recover\_all() {

185  cur\_block\_ = 0;

186  next\_loc\_ = blocks\_[0];

187  cur\_block\_end\_ = next\_loc\_ + sizes\_[0];

188  }

189

194  inline void start\_nested() {

195  nested\_cur\_blocks\_.push\_back(cur\_block\_);

196  nested\_next\_locs\_.push\_back(next\_loc\_);

197  nested\_cur\_block\_ends\_.push\_back(cur\_block\_end\_);

198  }

199

203  inline void recover\_nested() {

204  if (unlikely(nested\_cur\_blocks\_.empty()))

205  recover\_all();

206

207  cur\_block\_ = nested\_cur\_blocks\_.back();

208  nested\_cur\_blocks\_.pop\_back();

209

210  next\_loc\_ = nested\_next\_locs\_.back();

211  nested\_next\_locs\_.pop\_back();

212

213  cur\_block\_end\_ = nested\_cur\_block\_ends\_.back();

214  nested\_cur\_block\_ends\_.pop\_back();

215  }

216

222  inline void free\_all() {

223  // frees all BUT the first (index 0) block

224  for (size\_t i = 1; i < blocks\_.size(); ++i)

225  if (blocks\_[i])

226  free(blocks\_[i]);

227  sizes\_.resize(1);

228  blocks\_.resize(1);

229  recover\_all();

230  }

231

242  size\_t bytes\_allocated() {

243  size\_t sum = 0;

244  for (size\_t i = 0; i <= cur\_block\_; ++i) {

245  sum += sizes\_[i];

246  }

247  return sum;

248  }

249

250  };

251

252  }

253 }

254 #endif

stan::memory::stack\_alloc::bytes\_allocated

size\_t bytes\_allocated()

Return number of bytes allocated to this instance by the heap.

**Definition:** stack\_alloc.hpp:242

stan::memory::stack\_alloc::free\_all

void free\_all()

Free all memory used by the stack allocator other than the initial block allocation back to the syste...

**Definition:** stack\_alloc.hpp:222

stan::memory::stack\_alloc

An instance of this class provides a memory pool through which blocks of raw memory may be allocated ...

**Definition:** stack\_alloc.hpp:76

stan::agrad::sum

fvar< T > sum(const Eigen::Matrix< fvar< T >, R, C > &m)

**Definition:** sum.hpp:14

unlikely

#define unlikely(x)

**Definition:** likely.hpp:9

stan::memory::stack\_alloc::~stack\_alloc

~stack\_alloc()

Destroy this memory allocator.

**Definition:** stack\_alloc.hpp:149

likely.hpp

stan::memory::stack\_alloc::alloc

void \* alloc(size\_t len)

Return a newly allocated block of memory of the appropriate size managed by the stack allocator...

**Definition:** stack\_alloc.hpp:168

stan::math::size

int size(const std::vector< T > &x)

**Definition:** size.hpp:11

stan::memory::is\_aligned

bool is\_aligned(T \*ptr, unsigned int bytes\_aligned)

Return true if the specified pointer is aligned on the number of bytes.

**Definition:** stack\_alloc.hpp:32

stan::memory::stack\_alloc::start\_nested

void start\_nested()

Store current positions before doing nested operation so can recover back to start.

**Definition:** stack\_alloc.hpp:194

stan::memory::stack\_alloc::recover\_nested

void recover\_nested()

recover memory back to the last start\_nested call.

**Definition:** stack\_alloc.hpp:203

stan::memory::stack\_alloc::stack\_alloc

stack\_alloc(size\_t initial\_nbytes=DEFAULT\_INITIAL\_NBYTES)

Construct a resizable stack allocator initially holding the specified number of bytes.

**Definition:** stack\_alloc.hpp:133

stan::memory::stack\_alloc::recover\_all

void recover\_all()

Recover all the memory used by the stack allocator.

**Definition:** stack\_alloc.hpp:184


---

[ Stan Home Page ]

*© 2011–2014,
Stan Development Team.*
